# A miRNA target network putatively involved in follicular atresia

**DOI:** 10.1016/j.domaniend.2016.08.002

**Published:** 2017-01

**Authors:** F.X. Donadeu, B.T. Mohammed, J. Ioannidis

**Affiliations:** The Roslin Institute and Royal (Dick) School of Veterinary Studies, University of Edinburgh, Midlothian EH25 9RG, UK

**Keywords:** miRNAs, Follicle, Follicle atresia, Bovine, Granulosa, Theca

## Abstract

In a previous microarray study, we identified a subset of micro RNAS (miRNAs), which expression was distinctly higher in atretic than healthy follicles of cattle. In the present study, we investigated the involvement of those miRNAs in granulosa and theca cells during atresia. Reverse Transcription-quantitative Polymerase Chain Reaction (RT-qPCR) confirmed that miR-21-5p/-3p, miR-150, miR-409a, miR-142-5p, miR-378, miR-222, miR-155, and miR-199a-5p were expressed at higher levels in atretic than healthy follicles (9–17 mm, classified based on steroidogenic capacity). All miRNAs except miR-21-3p and miR-378 were expressed at higher levels in theca than granulosa cells. The expression of 13 predicted miRNA targets was determined in follicular cells by RT-qPCR, revealing downregulation of *HIF1A*, *ETS1*, *JAG1*, *VEGFA*, and *MSH2* in either or both cell types during atresia. Based on increases in miRNA levels simultaneous with decreases in target levels in follicular cells, several predicted miRNA target interactions were confirmed that are putatively involved in follicular atresia, namely miR-199a-5p/miR-155-*HIF1A* in granulosa cells, miR-155/miR-222-*ETS1* in theca cells, miR-199a-5p-*JAG1* in theca cells, miR-199a-5p/miR-150/miR-378-*VEGFA* in granulosa and theca cells, and miR-155-*MSH2* in theca cells. These results offer novel insight on the involvement of miRNAs in follicle development by identifying a miRNA target network that is putatively involved in follicle atresia.

## Highlights

• A subset of miRNAs are expressed at higher levels in atretic than healthy follicles.
• Most such miRNAs are expressed at higher levels in theca than granulosa cells.
• These miRNAs cooperatively regulate the expression of target genes during atresia.

## 1 Introduction

The overwhelming majority of follicles recruited from the primordial pool during a female's reproductive life will undergo atresia before they can reach the ovulatory stage. Atresia is an active process involving not only cell death but also resorption of follicular tissue and its replacement by stromal and new follicular tissue; these processes involve infiltration by immune and other cells, very much resembling wound healing [1]. Among several key regulators of wound healing and tissue remodeling processes across body tissues are microRNAs (miRNAs) [2].

The involvement of miRNAs in different aspects of follicle development has been demonstrated in numerous studies [3], [4]. Much of the existing evidence has been obtained using follicular cell cultures, mostly granulosa cells. Often reported effects of miRNAs include either the promotion or suppression of granulosa cell apoptosis [5], [6], [7], [8], [9], [10]. Yet, in many cases, the site of expression, if any, of these miRNAs within follicles (ie, granulosa and theca compartments) or whether their expression actually changes during follicle atresia, supporting their physiological role, has not been clarified. Moreover, although the posttranscriptional effects of miRNAs in tissues often involve targeting of a common gene simultaneously by several miRNAs and, at the same time, a single miRNA can simultaneously target multiple genes, previous functional studies in follicles have often used a one miRNA–one target approach thus providing limited information on the wider biological effects of miRNAs expressed simultaneously acting in coordination.

In a previous study, we used microarray to profile miRNA expression across a wide range of antral follicle development stages in cattle [11], a species which follicular physiology closely resembles the human, particularly when compared with rodents; by comparing miRNA profiles between steroidogenic-active and steroidogenic-inactive follicles, we identified a subset of miRNAs that are putatively involved in the growth of healthy dominant follicles. In the present study, we focused our attention on those miRNAs identified as upregulated during follicle atresia in our previous study. Specifically, we established and compared the expression of miRNAs and their putative targets within the follicular granulosa and theca compartments to gain insight into their involvement in follicle atresia.

## 2 Materials and methods

### 2.1 Collection and processing of bovine tissues

Follicles from ovaries of cycling beef cattle obtained at an abattoir were collected as part of a separate study [11]. Individual follicles 9–17 mm in diameter were dissected out, and the follicular fluid aspirated and centrifuged at 800 *g* for 10 min. The resulting supernatant was stored at −80°C until further analyses, and the cell pellet was combined with the follicular wall free of surrounding stroma and snap-frozen in liquid nitrogen until RNA extraction. Alternatively, after hemidissection, follicles were gently scraped with blunt-ended forceps to collect granulosa and theca wall compartments. Theca walls were washed repeatedly to remove any residual granulosa cells. Theca and granulosa cells from each individual follicle were then separately snapped frozen in liquid nitrogen.

Intrafollicular concentrations of estradiol and progesterone were measured using competitive double antibody radioimmunoassay kits (Siemens Healthcare Diagnostics Inc) following the manufacturer's instructions. All assays were validated in our laboratory by showing parallelism between serial sample dilutions and the provided assay standard curve. Sensitivity of the assays was 0.56 ng/mL and 0.01 ng/mL, and the intra-assay coefficient of variations were 6% and 4.3% for estradiol and progesterone, respectively.

### 2.2 RT-qPCR

Total RNA was extracted using the miRNeasy Mini kit (Qiagen, UK) and reverse-transcribed using the miScript II RT kit (Qiagen), as described [11]. Messenger RNA levels were quantified using the SensiFAST SYBR Lo-ROX Kit (Bioline Reagents Ltd, UK) and bovine-specific primers (Table 1). For miRNA quantification, the miScript SYBR Green PCR kit and miScript Primer Assays (Qiagen) were used. All PCRs were run on a MX3005P QPCR system (Stratagene, CA) using a standard curve to calculate copy numbers from Cq values [11]. Messenger RNA data were normalized using *18S* values within each sample, and miRNA data were normalized using endogenous *RnU6-2*. Mean intra-assay coefficient of variations for miRNA and mRNA qPCRs were 9.5% and 11.3%, respectively.

### 2.3 Micro RNA target identification

Identification of putative miRNA targets was done using miRTarBase release 6.0 and TargetScan release 7.0 to select targets experimentally validated in human and/or rodents (by reporter assay, Western blot and/or RT-qPCR, as detailed in http://mirtarbase.mbc.nctu.edu.tw/) and computationally predicted targets within the bovine genome (http://www.targetscan.org), respectively. For convenience, each identified miRNA target interaction was classified as high-, medium-, or low-confidence based on whether it was present in both miRTarBase and TargetScan, miRTarBase only or TargetScan only.

### 2.4 Statistical analyses

Robust regression and outlier removal (ROUT) test was applied to datasets, and outlier values (*P* < 0.01) were excluded from subsequent analyses. Gene expression data were assessed for normality using the D'Agostino and Pearson normality test and were log-transformed before statistical analysis where necessary. Two-way analysis of variance followed by unpaired *t*-tests to identify differences in gene expression between healthy and atretic follicles within each cell type were used. Significance was considered at *P* < 0.05, whereas differences with *P* values <0.1 were considered to approach significance. Nomenclature according to miRBase release 21 is used throughout the manuscript. All miRNAs referred to are bovine (bta-) except otherwise specified.

## 3 Results

### 3.1 Micro RNA expression analyses in follicular tissues

**Fig. 1:**
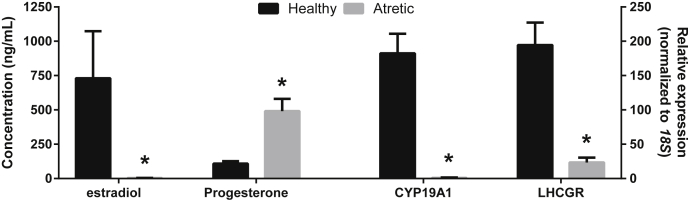
Mean (± standard error) concentrations of estradiol, progesterone, and transcript levels of *CYP19A1* and *LHCGR* in bovine follicles (9–17 mm) classified as healthy (n = 26) or atretic (n = 15). Differences between group means are indicated by asterisks (*P* ≤ 0.05).

In a previous study in cattle [11], microarray analyses yielded a total of 11 unique bovine sequences, which were expressed in greater abundance (>1.5-fold) in atretic than in healthy preovulatory size follicles (Table 2). The status of the follicles analyzed in that study had been predetermined on the basis of steroidogenic capacity and *LHGCR* expression in granulosa cells (Fig. 1). Microarray results were validated by RT-qPCR in the present study, confirming the upregulation of 9 miRNAs in atretic follicles (Table 2).

**Fig. 2:**
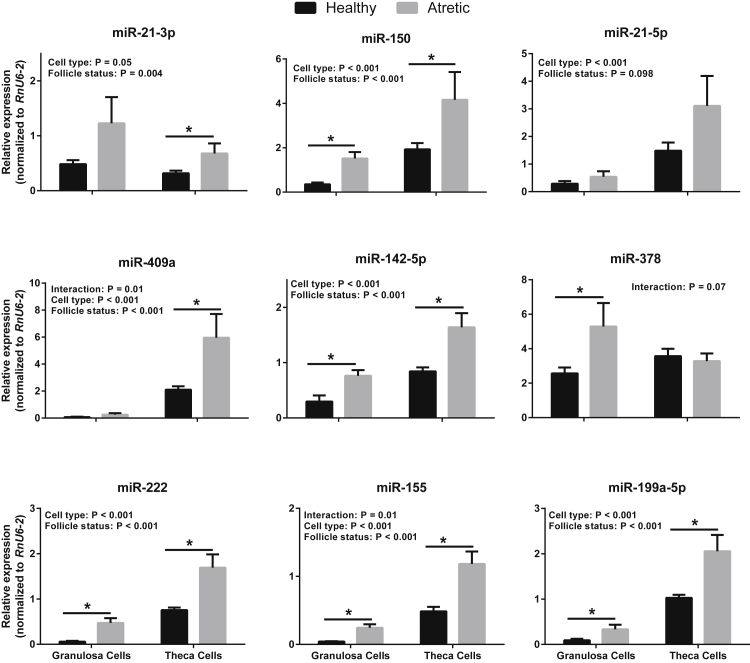
Relative miRNA levels (mean ± standard error; normalized to levels of *RnU6-2*) in granulosa and theca cells from bovine healthy (n = 9) and atretic (n = 6) follicles. Differences between group means within each cell type are indicated by an asterisk (*P* < 0.05). miRNA, micro RNA.

To gain insight on the involvement of these miRNAs in follicle development, we quantified their relative expression in granulosa and theca cell compartments. This showed that all miRNAs except for miR-21-3p and miR-378 were expressed in greater abundance (between 2-fold and 25-fold) in theca than in granulosa cells (cell type, *P* < 0.05; Fig. 2). Moreover, an effect of follicle status (*P* < 0.05), owing to overall higher expression levels in atretic follicles, was detected for all miRNAs except miR-21-5p and miR-378; for miR-378, an interaction approached significance (*P* = 0.07), reflecting higher expression levels in granulosa but not theca cells from atretic follicles.

### 3.2 Identification and expression analyses of miRNA targets

**Fig. 3:**
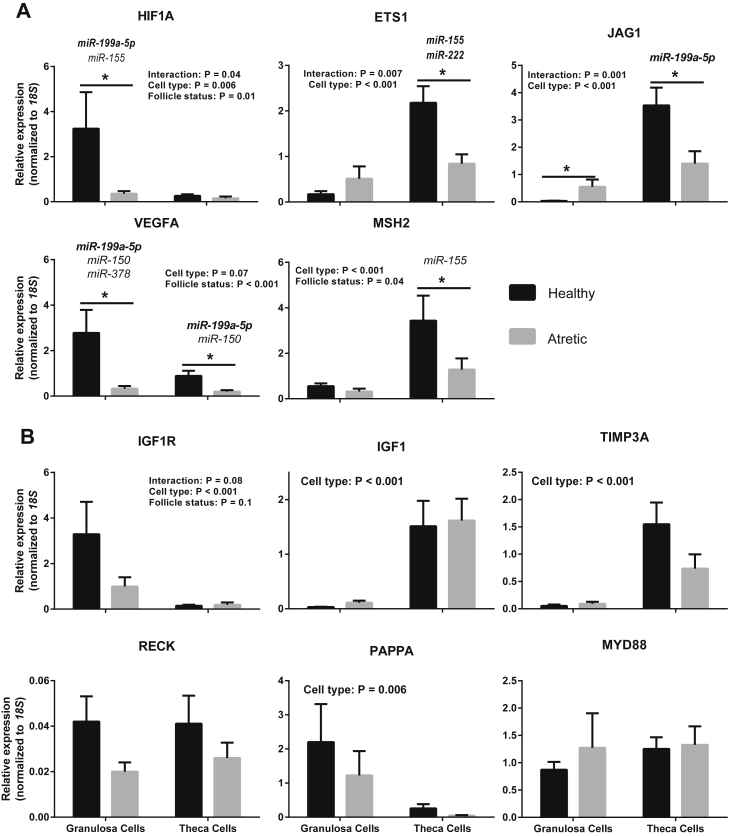
Relative mRNA levels (mean ± standard error; normalized to levels of *18S*) in granulosa and theca cells from bovine healthy (n = 9) and atretic (n = 6) follicles. Genes confirmed as miRNA targets by PCR are shown in (A), other genes are shown in (B). Putative targeting miRNAs are shown on top of the corresponding graph bars in (A); all indicated miRNA target interaction were obtained from miRTarBase (database of experimentally validated miRNA target interactions in human and/or rodents), and interactions that were in addition computationally predicted in cattle (obtained from TargetScan) are indicated by miRNAs in bold (eg, miR-199a-5p-*HIF1A*). Differences between group means within each cell type are indicated by an asterisk (*P* < 0.05). mRNA, messenger RNA; miRNA, micro RNA.

To identify putative targets of the 9 miRNAs in cattle, we used miRTarBase, a database containing experimentally validated targets of human, mouse, and rat miRNAs, and we selected 36 genes that were simultaneous targets of ≥2 of those miRNAs (Table S1). To increase confidence in our target selection, we then searched all 36 genes in TargetScan and selected those, 16 in total, which bovine homologues were computationally predicted targets of one or more of the corresponding bovine miRNA sequences (Table S1). Finally, we assessed the validity of these predictions by analyzing by qPCR the expression in follicular cells of 8 of the 16 genes identified (Table 3 and Fig. 3; an additional 2 targets, *E2F2* and *SIRT1*, were also selected but could not be detected in follicular cells by RT-qPCR). For completeness, our qPCR analyses also included 1 gene (*MYD88*), which interactions with 2 miRNAs were experimentally validated but not computationally predicted in bovine (Table 3 and Fig. 3), and 2 genes, *IGF1* and *PAPPA*, which were computationally predicted bovine targets of 2 miRNAs (miR-222 and miR-378) and 3 miRNAs (miR-142-5p, miR-150, and miR-378), respectively, but none of which were experimentally validated in any species, ie, they were present in TargetScan but not in miRTarBase (Table 3 and Fig. 3).

As shown in Figure 3, of the remaining miRNA targets, 8 were enriched (cell type, *P* < 0.05) in either granulosa cells (*HIF1A*, *IGF1R*, and *PAPPA*) or theca cells (*ETS1*, *JAG1*, *MSH2*, *IGF1*, and *TIMP3A*), and 5 targets were differentially expressed according to follicle status, in all cases involving a reduction in atretic follicle cells, as indicated by a significant effect of follicle status or a follicle status × cell type interaction (*HIF1A*, *ETS1*, *JAG1*, *VEGFA*, and *MSH2*).

### 3.3 Validation of miRNA target interactions

Comparing expression profiles between miRNAs and mRNAs (Fig. 2, Fig. 3) allowed for the testing, based on a negative association between the expression of a miRNA and that of its predicted target(s) within a follicular cell type, a total of 12 high-confidence miRNA target interactions (ie, obtained from both miRTarBase and TargetScan; indicated by dark gray in Table 3), 8 medium-confidence interactions (obtained from miRTarBase but not present in TargetScan; indicated by light gray in Table 3), and 5 low-confidence interactions (identified in TargetScan but not present in miRTarBase; indicated by an “X” in Table 3).

Of the 12 predicted high-confidence interactions analyzed, 5 were confirmed by RT-qPCR, specifically involving miR-199a-5p and *HIF1A* in granulosa cells, miR-155/miR-222 and *ETS1* in theca cells, miR-199a-5p and *JAG1* in theca cells, and miR-199a-5p and *VEGFA* in both granulosa and theca cells (miRNAs indicated in bold in Fig. 3A). For another 3 predicted high-confidence interactions, differences in mean miRNA and target levels did not reach significance (*P* < 0.1). These involved *JAG1* and miR-21-5p in theca cells, *MSH2* and miR-21-5p in theca cells, and *IGF1R* and miR-378 in granulosa cells (Fig. 3A,B). Four predicted high-confidence interactions involving *TIMP3* and *RECK1* were not confirmed as the levels of these transcripts did not change significantly according to follicle status (Fig. 3B).

Of 8 predicted medium-confidence interactions analyzed (Table 3), 4 were confirmed involving miR-155 and *HIF1A* in granulosa cells, miR-150 and *VEGFA* in both granulosa and theca cells, miR-378 and *VEGFA* in granulosa cells, and miR-155 and *MSH2* in theca cells (Fig. 3A). For another 2 medium-confidence interactions, involving miR-21-5p and *VEGFA* in both granulosa and theca cells, and *IGF1R* and miR-21-5p in granulosa cells (Fig. 3A,B), differences in mean miRNA and target levels did not reach significance (*P* < 0.1), whereas the remaining 2 medium-confidence interactions were not confirmed as the levels of *MYD88* did not change with follicle status (Fig. 3B).

Finally, none of the 5 low-confidence interactions (Table 3) tested were confirmed as transcript abundance of *IGF1*, and *PAPPA* did not change according to follicle status (Fig. 3B).

## 4 Discussion

A limited number of studies in cattle [11], [12] and pigs [8] have reported genome-wide miRNA expression profiles associated with follicle atresia. Five of the 9 miRNAs confirmed to be upregulated in atretic follicles in the present study (miR-21-5p, miR-21-3p, miR-222, miR-155, and miR-199a-5p) were also found to be increased in subordinate relative to dominant follicles on day 3 of the bovine estrous cycle using deep-sequencing rather than microarray [12]. Another miRNA, miR-378, was previously shown, together with miR-21-5p, to increase in expression in subordinate and anovulatory follicles in horses [13], [14]. Taken together, these results are consistent with an involvement of these miRNAs in follicular atresia in the monovular ovary. Indeed, all 9 miRNAs identified in atretic follicles in this study can reportedly regulate cell survival and/or tissue turnover [15], [16], [17], [18], [19], [20], [21]. Specifically in the ovary, miR-21 promotes cell survival during luteinization [10], whereas at the same time it is expressed at very high abundance in the regressing corpus luteum [22], suggesting a multifaceted, developmental stage–dependent involvement in follicle and corpus luteum function. A putative involvement of miR-378 in regulating luteal cell survival in bovine has been suggested but not proven [23]. However, in the pig, miR-378 targets aromatase and progesterone receptor in granulosa cells and regulates both ovarian estradiol production and oocyte maturation [24], [25], [26]. Finally, another of the miRNAs investigated in our study, mir-222, may reportedly regulate steroidogenesis of granulosa cells [27].

**Fig. 4:**
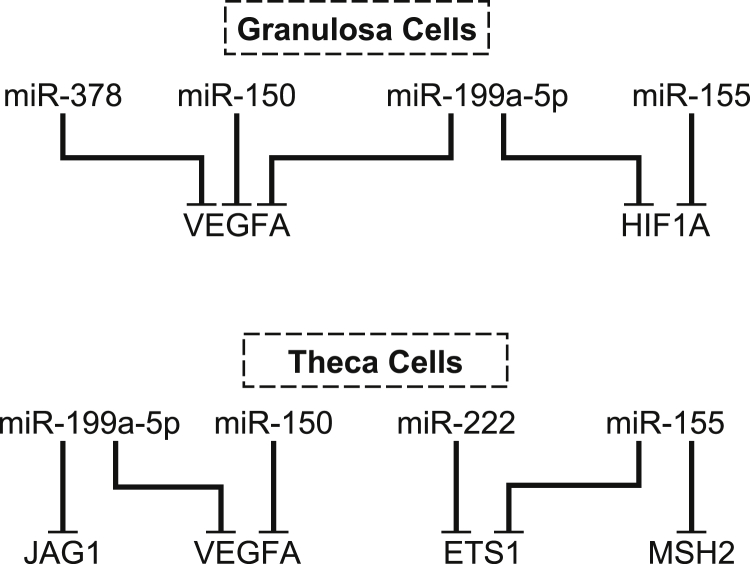
Schematic summary of putative miRNA target interactions in granulosa and theca cells during follicular atresia identified in this study. miRNA, micro RNA.

Our miRNA target pair analyses provides novel insight on the molecular regulation of follicular atresia by identifying specific miRNA networks putatively involved within different follicular compartments (summarized in Fig. 4). To identify high-confidence bovine miRNA targets, we selected genes that both (1) had already been experimentally validated for 2 or more miRNAs in different cellular contexts in humans and/or rodents (as bovine-specific information is not available) and (2) contained predicted miRNA target sites in the bovine homolog 3′ UTR. In choosing this approach, we took into consideration that (1) effective target downregulation often involves multiple miRNAs simultaneously binding the 3′ UTR of a gene and (2) computational prediction of miRNA targets is relatively inaccurate in terms of both false targets being identified and true targets being missed. A similar proportion of predicted miRNA target interactions classified as high confidence (identified from both miRTarBase and TargetScan) and medium confidence (identified from miRTarBase only) were confirmed by qPCR (5 of 12 and 4 of 8, respectively), with another 3 and 2 interactions failing to be validated because miRNA and/or mRNA expression differences only approached significance (*P* < 0.1). These results highlight the notion that a significant number of true miRNA targets are normally missed using computational prediction approaches and that the fact that a target has been experimentally validated in other species may provide the strongest rationale for target selection, particularly considering that many miRNAs are functionally conserved. It needs to be pointed out that failure to detect differences in predicted target levels by RT-qPCR (eg, *MYD88* in this study) does never by itself provide conclusive evidence that the gene in question is not an actual target, as miRNAs may in general have greater effects on protein than transcript levels. Unfortunately, quantification of protein levels is not always possible in bovine due to the limited availability of species-specific antibodies.

Among the genes confirmed as miRNA targets in atretic follicles was *VEGFA*, which expression was significantly downregulated in both granulosa and theca cells, putatively through the effects of at least 3 different miRNAs. Within the follicle, VEGFA has strong trophic effects not only in vascular cells but also in steroidogenic cells [28], [29]. A key transcriptional activator of VEGFA is HIF1A [30]. This is a gonadotropin-induced, master regulator of cellular responses to hypoxia which expression in granulosa cells mediates survival, steroidogenic, and angiogenic responses within the follicle [30], [31]. HIF1A is reportedly expressed at higher levels in granulosa than theca within nonatretic follicles [32]. These observations are consistent with our finding of a simultaneous decrease in the expression of *HIF1A* and *VEGFA* during bovine follicle atresia, particularly in granulosa cells. Our results implicate a network of miRNAs, namely, miR-199a-5p, miR-155, miR-150, and miR-378, in the downregulation of the HIF1A-VEGF effector system during atresia, with one miRNA, miR-199a-5p, simultaneously targeting both genes, as reported in other cell types [33].

Two miRNA targets identified in theca cells and which, albeit much less characterized, may also be involved in angiogenesis, are the transcription factor, ETS1, and the NOTCH1 ligand, JAG1. ETS1 is a proto-oncogen highly expressed in immune and vascular cells. In the ovary, it has been shown to be expressed in theca and granulosa cells and its expression to dynamically change during the estrous cycle [34], [35]. An involvement of ETS1 in regulating RGS2 expression during ovulation has been shown [36]. Moreover, its role in promoting angiogenesis or as a proapoptotic factor in different cell types [37] could account for the changes in expression during follicle atresia. Likewise, apart from its reported role in promoting early follicle development, little information is available on the role of JAG1 in the ovary. An involvement of JAG1 in follicular angiogenesis is suggested by its expression in endothelial and other vascular mural cells in mouse ovaries [38]. The reason for the opposite trends in *JAG1* expression in granulosa and theca cells of healthy and atretic follicles in our study is unknown, warranting further study of the functions of this gene in the adult ovary.

*MSH2*, a gene involved in DNA mismatch repair, was confirmed as a target of miR-155 in theca cells. This is consistent with the finding in cattle that atresia involves the downregulation of cell cycle and DNA replication genes in theca cells rather than the downregulation of apoptotic genes as occurs in granulosa cells [39]. Interestingly, a study showed that another miRNA, miR-26a, targeted the cell cycle checkpoint kinase, *ATM*, in porcine follicular cells during atresia, leading to increased DNA breaking and apoptosis, and raising the possibility that miR-155 could exert a similar effect through targeting *MSH2* in bovine theca cells.

Finally, because of the heterogeneous nature of follicular tissue, particular theca, it is not possible to determine, from our data, the specific cell types involved in the identified miRNA target interactions. For example, although most of the miRNAs analyzed in this study are known not to be cell-specific, miR-150 and miR-155 are highly expressed and primarily regulate hematopoietic and vascular cells. Nonetheless, paracrine regulation of gene expression by miRNAs has also been described whereby miRNAs are produced by one cell type and then secreted to regulate gene expression of a different cell type within a tissue [40], adding further complexity to the role of miRNA target interactions during follicular atresia.

In summary, by establishing the expression patterns of miRNAs and their putative targets in granulosa and theca cells of healthy and atretic follicles in cattle, we have identified a network of miRNAs including miR-199a-5p, miR-155, miR-222, miR-150, and miR-378, which we propose are involved in follicle atresia through combined targeting of genes involved in cell survival, proliferation, and differentiation; namely *HIF1A* and *VEGFA* in granulosa cells and *MSH2*, *ETS1*, *JAG1*, and *VEGFA* in theca cells. Although the identified miRNA target interactions should be confirmed by gene targeting or other molecular approaches in future studies, our results will provide fertile ground for further hypothesis testing toward a better understanding of the molecular mechanisms involved in follicle atresia.

## Figures and Tables

Data adapted from Sontakke et al, 2014.

**Table 1:** Primer sequences used in messenger RNA analyses.

Gene	Sequence (5′-3′) sense/antisense
18S	GCTGGCACCAGACTTG/GGGGAATCAGGGTTCG
CYP19A1	CGCAAAGCCTTAGAGGATGA/ACCATGGTGATGTACTTTCC
E2F2	TCGCTATGACACATCGCTGG/CGTCACGTAGGCCAGTCTCT
ETS1	CACAGTCTCTCCGGCAAAGT/GTGGATGATAGGCCGACTGG
HIF1A	CAGAAGAACTTTTGGGCCGC/TCCACCTCTTTTGGCAAGCA
IGF1	AGTGCTGCTTTTGTGATTTCTTGA/GCACACGAACTGGAGAGCAT
IGF1R	AAGCTGAGAAGCAGGCAGAG/CGGAGGTTGGAGATGACAGT
JAG1	GAGTGTGAGTGTTCTCCGGG/TTGGCCTCGCATTCATTTGC
LHCGR	GGACTCTAGCCCGTAGG/ACACATAACCACCATACCAAG
MSH2	TGGGCAGAAGTGTCCATTGT/CCCACGCTAATCCAAACCCA
MYD88	AAGTTGTGCGTGTCTG/GGAAATCACATTCCTTGCT
PAPPA	TTGCTGCGCTTCTACAGTGA/GCACAGTCACCCTGTAGGTC
RECK	GTGCTTCCTTCTCTTGTCTGGA/GGCTTGACAGTATTCTCGGC
SIRT1	GCTTACAGGGCCTATCCAGG/TATGGACCTATCCGAGGTCTTG
TIMP3	GGATTCACCAAGATGCCCCA/GAGCTGGTCCCACCTCTCTA
VEGFA	TGTAATGACGAAAGTCTGGAG/TCACCGCCTCGGCTTGTCACA

**Table 2:** Bovine miRNA sequences which expression was upregulated (>1.5-fold) in atretic relative to healthy follicles^a^.

miRNA	Microarray		RT-qPCR	
	Fold change	Adjusted *P* value^b^	Fold change	*P* value
bta-miR-483/hsa-miR-483-3p	3.64	0.001	0.88	0.409
bta-miR-21-3p/hsa-miR-21-3p	3.09	0.002	3.38	0.021
bta-miR-150/hsa-miR-150-5p	2.54	0.001	2.92	0.001
bta-miR-21-5p/hsa-miR-21-5p	2.39	0.001	4.90	0.001
bta-miR-409a/hsa-miR-409a-5p	2.36	0.000	1.85	0.001
bta-miR-744/hsa-miR-744-5p	2.36	0.002	0.73	0.057
bta-miR-142-5p/hsa-miR-142-5p	2.03	0.001	2.81	0.001
bta-miR-378/has-miR-378a-3p	1.91	0.001	1.51	0.017
bta-miR-222/hsa-miR-222-3p	1.84	0.000	1.92	0.001
bta-miR-155/hsa-miR-155-5p	1.66	0.019	5.66	0.001
bta-miR-199a-5p/hsa-miR-199a-5p	1.63	0.004	1.66	0.001

a Microarray analyses were performed in 6 healthy and 5 atretic follicles (12–17 mm in diameter) in a previous study (data adapted from Sontakke et al, 2014). Microarray data were validated by qPCR in the present study using 26 healthy and 15 atretic follicles (9–17 mm in diameter).

b FDR; Benjamini and Hochberg adjustment.

**Table 3:** Candidate miRNA target interactions that were analyzed by qPCR (see Fig. 3).

Abbreviation: miRNA, micro RNA.

High-confidence miRNA target interactions obtained from both miRTarBase and TargetScan (ie, both have been experimentally validated in human, rat and/or mouse and are computationally predicted in bovine) are shown in dark gray. Medium-confidence interactions obtained from miRTarBase only are shown in light gray. Low-confidence interactions obtained from TargetScan only are indicated by an “X”.
